# Virtual Dye Angiography: flow visualization for MRI-guided interventions using endogenous contrast

**DOI:** 10.1186/1532-429X-13-S1-O25

**Published:** 2011-02-02

**Authors:** Ashvin K George, Anthony Z Faranesh, Kanishka Ratnayaka, JA Derbyshire, Robert J Lederman, Michael S Hansen

**Affiliations:** 1 NIH, Bethesda, MD, USA

## Objective

To efficiently visualize blood flow in real-time without exogenous contrast agents.

## Background

Visualizing blood flow is important in MR-guided interventions (e.g. repairing structural heart defects). We introduce a method, called Virtual Dye Angiography (VDA), to visualize flow in a manner inspired by contrast-enhanced X-ray angiography. Slightly similar to arterial spin labeling, VDA saturates a localized region using multidimensional RF pulses. Unlike phase-contrast velocity mapping, which has unsuitably long acquisition times, VDA can be integrated into existing high-contrast, high-SNR SSFP imaging sequences with minimal modification.

## Methods

Porcine experiments were performed on a 1.5T scanner (Siemens;Espree) using a real-time multi-slice SSFP sequence that was modified to include VDA. When VDA was enabled (in real-time) a cylindrical column (whose size and location were interactively controlled) was continuously saturated. The VDA module consisted of a shaped RF pulse played concurrently with a spiral gradient[1] (to flip spins in the cylindrical column into the transverse magnetization plane) followed by a spoiler gradient in the slice direction (to saturate the column). Two slices (1 and 2) were imaged in real-time with the VDA saturation module (13 ms) played before the acquisition of, and with the saturation column perpendicular to, slice 1.

## Results

**Figure 1:**
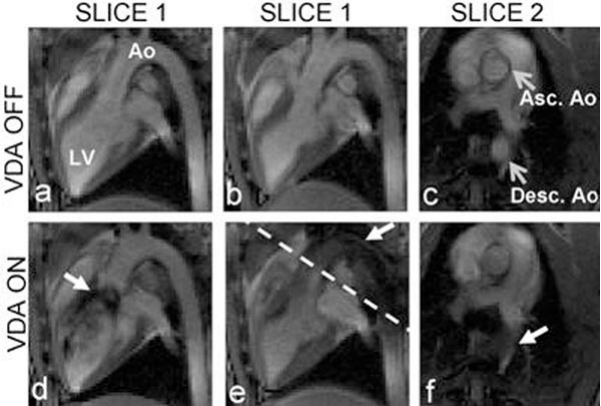
LV to Ao flow

**Figure 2:**
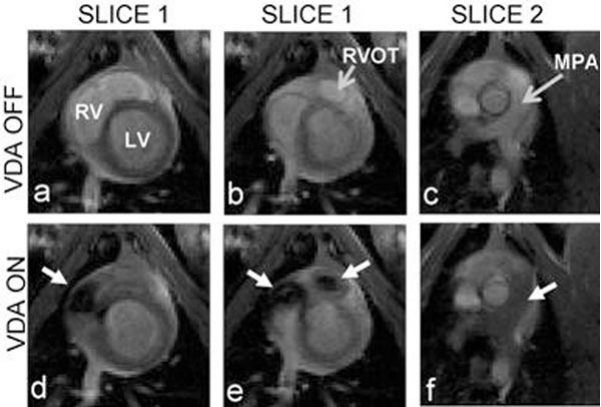
RV to MPA flow

**Figure 3:**
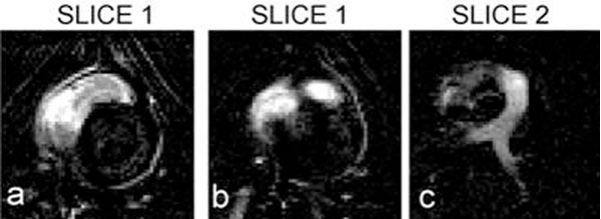
Subtraction images corresponding to Figure 2

VDA was demonstrated in multiple locations in the pig heart. Figure 1 shows flow from the LV to the aorta; without VDA (top row) and with VDA (bottom row). The dashed white line in (e) denotes the relative position of slice 2 to slice 1. The saturation column is placed through the LV. The white arrows track the saturated spins: in initial saturation in the LV (d) and into the aorta (e and f). Figure 2 shows flow from the RV to the pulmonary vessels using a saturation column placed through the RV. The white arrows track the saturated spins: in initial saturation in the RV (d), then exiting the RV outflow tract (e), and in the pulmonary arteries (f). Figure 3 shows the corresponding subtraction images.

## Conclusions

We have introduced a real-time method of visualizing blood flow. Its modularity allows for minimal modification of the existing sequence. Future refinements we envision include cardiac synchronization and motion compensation for consistent placement of the saturation column and the use of DSA-like image subtraction for enhanced visualization.
